# The Use of the Pyrometer in Fusing Porcelain

**Published:** 1906-09-15

**Authors:** R. M. Pelton


### THE USE OF THE PYROMETER IN FUSING PORCELAIN.

BY R. M. PELTON, D.D.S.

Read before the Michigan Dental Association, July 10, 1905.

The purpose of this paper is to endeavor to make clear the proper use and application of a Pyrometer in the fusing of porcelain.

It might be supposed that all that is necessary aside from the ordinary knowledge of porcelain work is to connect the pyrometer to the furnace and proceed to make tests upon the various bodies that are to be used, observing the length of time taken and the degree of heat to which the needle points when the desired result is obtained, making a note of it mentally or otherwise for future guidance and ever afterwards proceed in the same manner.

This might be ample directions in the case of a steady voltage but the fact is that current strength varies in some localities, and this variation causes trouble under any system which may be adopted for the fusing of porcelain.

Therefore a further consideration of the subject is necessary to thoroughly understand it and to enable the operator to account for certain phenomena he will observe when using a pyrometer on a fluctuating current. Phenomena which at first may not be understood, will likely be attributed to other causes than the real one.

Before entering upon that subject it will be necessary in order to understand it more clearly to consider some phenomena about porcelain that have not long been observed, at least nothing was said about it in any published reports until the matter was taken up by Dr. John Q. Byram of Indianapolis, in a very instructive and interesting paper, read before the Chicago Odontographic Society November 21, 1905. The statements made by the doctor at that time were based upon a very thorough series of experiments and the results he obtained have been verified by others. Some of the deductions from these experiments are as follows:

1st.—That porcelain has no definite fusing point when separated from the consideration of time.

2nd.—The longer the time of exposure to heat of any porcelain the lower the temperature at which it will fuse. To quote Dr. Byram exactly, "By prolonging the time of exposure to heat, a thoroughly fused porcelain may be obtained at a comparatively low temperature."

3rd.—That low fusing porcelains may be obtained by repeated fusing and grinding of high fusing porcelain.

4th.—If a piece of porcelain be fused perfectly and then more of the same porcelain be added and the whole mass carried to exactly the same point, the first layer will be over fused because it has had a longer exposure to the same heat. If a third layer be added, and fused perfectly the second will be overfused and the first still more so and the colors bleached. In making this test layers may be added and the heat carried to the exact fusing point of the last layer until by the repetition the first is absolutely ruined or in other words if porcelain be kept long enough in the maximum heat required to fuse it, it will become just a globule of glass.

This quality in porcelain which admits of its fusion at a comparatively low temperature by long exposure is so pronounced that those which ordinarily fuse at from about 2100 to 2550 degrees when brought to that temperature rapidly, may be fused on pure gold by giving them sufficient time. Pure gold fuses at a trifle under 1950 degrees, and Dr. Byram's experiments show that Brewster's enamel could be properly fused on it in from 15 to 45 minutes, the difference in time depending upon the amount of heat applied. Brewster's foundation, Justi's and Close's body require from 30 minutes to one hour and fifteen minutes. Brewster's Crown and Bridge, S. S. White's inlay, and Whiteley's inlay porcelains require from one to three hours, and the Consolidated High Fusing inlay porcelain requires from six to eight hours to fuse on pure gold.

From the foregoing we see that by repeated heating of porcelain to say the point of biscuiting will, in a few heatings produce a perfect fuse, or if porcelain be exposed to a certain degree of heat for a certain length of time, accurate and desired results will follow which will be known in advance by the operator who has made the necessary tests. Success depends upon time and temperature intelligently applied.

The time is easily disposed of, but what about the temperature. How are we to fuse at a definite degree of heat without some means for indicating it. Some may say we can judge by the color of the heat, but that method is unreliable to say nothing of the terrible strain upon the eyes when using high fusing bodies. Another may say leave the furnace running with the lever on No. 1 until the maximum heat is obtained on that button, and then always start from that and time the work on the other points used until the proper result is reached. This gives us a definite heat although we do not know what degree it is. That may be true on a steady voltage and we know an immense amount of the most excellent work has been done with this method, but what about the dangers of a fluctuating current? In this case if time only is depended upon to obtain correct results, the situation becomes at once serious when we consider the danger of running perhaps, an important piece of work that has taken hours to bring to the point where it is ready for the furnace. Let us presume a case: Yesterday we obtained correct results by running, say half minute on contacts, No. 2, 3, 4, and 5, and 2 minutes on 6, and today we again ran the furnace in exactly the same manner, but obtained only a biscuit where yesterday a perfect fuse was the result. What is the meaning? As we all know it means that today the voltage is weaker than it was yesterday, and we must test up again. Perhaps no harm is done in this case, but tomorrow if we depend on the tests of today, it may be the voltage will be as strong or stronger than yesterday and if we run the length of time indicated by our tests of today, the work may be ruined and require to be all done over again.

Under tests with the pyrometer if the current is uniformly the same day after day, the needle will always point to the same temperature upon running the same length of time. The results to porcelain will be the same with each fuse if approximately an equal amount of the powdered body be taken, and the only responsibility involved is to decide upon the allowance to be made for the difference in amount of porcelain which would be necessary under any system of fusing, and to move the leaver in the usual lengths of time on the intermediate contacts from one to that which is decided upon for the final result. The pyrometer may be relied upon, and it also serves another purpose of great value. It indicates when the desired point is reached and also enables the operator to readily observe any serious fluctuation of heat from that which should be produced under the steady voltage, for if, from any cause the current should be either weaker or stronger than normal, the needle will soon denote the resulting variation in amount of the heat by moving to a greater or lesser distance on the dial than is usual in the same length of time, and this change will instantly be noted, the cause of it understood, and the proper steps taken to avoid the damage to the work.

The steps should be three in number and the decision as to the proper one to take, and how far it should be carried, will be based upon experience.

Step No. 1.—If the needle indicated that heat was being generated more slowly than usual, the observing operator would know he must take a longer time for the fuse, and as the porcelain would thereby receive a longer exposure to heat, it would fuse at a lower temperature. Therefore the needle would not be allowed to register so high as when the voltage and resulting heat is normal.

Step No. 2.—On the other hand if the needle indicated that heat was being generated more rapidly than usual he would know that if the lever be moved forward as usual the fuse will be reached in a shorter time and as the exposure of the porcelain to heat is for a lesser time, the needle must be allowed to indicate a higher temperature than when the voltage and heat is normal.

Step No. 3—If the operator observes that the needle is registering a greater or less amount of heat in a given time than usual, he may still hold his work to the normal conditions by moving the rheostat lever more rapidly or slowly as required to reach a given temperature in a given time. This step is very likely the best of all and incidentally is a good argument in favor of a rheostat.

Some may think all this might not be noted but it would be a mistake to suppose any thing of the kind. It becomes second nature to the careful operator to be conscious of the doings of the needle and the degrees to which it should point in a given time. For instance, if we start with the normal heat obtained by leaving the furnace running on No. 1 contract, with the door open, and then close door and place lever on No. 2 remaining there 30 seconds and the needle points to 1550 today at the end of that 30 seconds and tomorrow under apparently the same conditions the needle points to 1660 in in the same length of time, the operator would be conscious of it and know that his current was stronger, and if he adopted the plan of regulating to the changed condition by use of the rheostat, he would advance the lever more slowly and hold the muffle to normal conditions.

The Pyrometer needle will point to the same degree of heat every time when pure gold fuses, if the gold has the same bulk, and is in exactly the same temperature as the hot end of the Thermo-Couple every time the fuse is made, and this is regardless of the length of time taken.

Assuming that the instrument has been correctly calibrated it will always give the correct temperature of the muffle. When heat is generated rapidly in a muffle, a short run will fuse the porcelain but it will be at a comparatively high heat and the needle correctly indicates what degree it is.

On a comparatively low heat and the necessity of a long run the same porcelain will eventually fuse, it may be several hundred degrees lower but the temperature will be registered by the pyrometer and it will be correctly reckoned from the fusing point of pure gold as the standard.

With this array of proven facts, unless what seems very improbable takes place, and the nature of porcelain in reference to its fusing qualities is entirely changed, so that it approaches more nearly that of gold, it does not seem possible that any automatic device operated by the fusing of metals will ever be invented that can be relied upon to any extent to cut off the current at the proper time, while the operator busies himself with something else or closes up and leaves his office entirely.

Neither is it reasonable to suppose that any man can write names of the various porcelain bodies on the dial of a pyrometer and with any degree of certainty say that such a porcelain will fuse here and another there under the varying conditions of current which are sure to be met with.

If a pyrometer be calibrated with the Thermo-Couple attached to a furnace which is heating on a 110 volt current, and after calabration a porcelain be fused, say at 2400, then attach the same outfit to a 120 Volt current, the extra 10 volts will generate heat more rapidly and the porcelain be fused in a shorter time, therefore it must be in a higher heat. In this case it would be at least 100 degrees. Again if the same outfit were attached to a 104 volt current the reverse would occur, and from the slowly generated heat and longer time the needle would point to a lower temperature than 2400. In the three cases the calibration would be the same, on gold with its definite fusing point, and in all three, the heat indicated by the needle would be correct.

In most localities where the voltage is between 104 and 125 it is given as 110, but it varies from that. Here in Detroit in the downtown district and on the Edison current, the voltage is from 118 to 120 direct. In the power buildings here and elsewhere, where the current is generated in the buildings, there is usually considerable fluctuation on account of the varying loads put upon it. In a large portion of Detroit the current is 109 alternating. This point is mentioned to show how difficult it is to lay down fixed rules, and to make more clear the necessity for each dentist to make his own tests on his own current and with the porcelain outfit he has elected to use. He can then write down fusing points as he finds them under conditions with which he soon becomes familiar.

There is something definite and tangible about a Pyrometer, a something to rely on and give a name to. The time and degrees can be thought about and talked about and the tests will be at once fixed in the mind in a way that is very gratifying and the operator is relieved from ever straining the eyes in attempting to look into the fierce heat of the muffle.

To the beginner in porcelain work who wishes to use a pyrometer, we would advise the fusing of porcelain buttons, making careful tests, and at the same time observing temperature indicated by the needle and the time occupied, make comparisons of color, bearing in mind that in proportion as the button is smaller the shade will be lighter than that of the shade guide. Do not try to obtain the maximum heat supposed to be required at first, rather get an underfuse and then work up to the correct results. Do not mistake the porous burned out appearance of an over fuse for an under fuse, and then put a useless strain on your appliance trying to make it come out right. This very common mistake among beginners is quite surely avoided in the use of the pyrometer, as they are given warning by the point to which the needle advances. With this system as with any other no practical case should be attempted without this preliminary testing and these tests should include various kinds of work before attempting any thing for a patient.

If it is desired, and should your current prove to be very uniform, good results may be obtained by omitting to observe the time and in its place move the lever forward when the needle reaches a certain point, which is equivalent to timing, as experience has shown that the needle arrives at that point in a given time. The same holds true on the final point selected for the fuse in the case of a steady current, for it will be known that when the needle indicates a certain degree of temperature it has arrived there in the usual time and the porcelain has been exposed to the exact heat for the proper length of time for its fusing.

In using a pyrometer in connection with continuous gum work the fact is at once brought out that the fuse is obtained ordinarily at a much lower temperature then in small work because a long time is usually taken to do it. It is very likely the general belief that on a long run with the continuous gum furnace the fuse is finally obtained at the same degree of heat as a short run in the small furnace, but this is a mistake.

In Toronto last year, I placed a button of Consolidated body in the furnace and closed the door with the lever on No. 1 contact. Just then I was called away, and was gone for a considerable length of time, and when I came back, I took out the body, and to my suprise, it was beautifully fused. I supposed then that the long run on No. 1 with the door closed had brought the temperature up to the fusing point of that body which is given as 2550 degrees Fahr. I now know my mistake for by the pyrometer I can show that under the conditions in which the furnace was working at the time it would be impossible to obtain over 2100 degrees with the door closed and the lever on No. 1, no matter how long it should be left. So I knew *that* porcelain fused at 2100 or less.

I have tried to bring out this point strongly because it will, perhaps, be the only one that may puzzle the man who takes up the pyrometer methods. The fact exists and it was shown up when the Pyrometer came into use and gave us a tangible method of indicating degrees of heat.

